# Cytotoxicity Profiles for a Series of Triorganophosphinegold(I) Dithiocarbamates and Triorganophosphinegold(I) Xanthates

**DOI:** 10.1155/S156536330400010X

**Published:** 2004

**Authors:** Dick de Vos, Soo Yei Ho, Edward R. T. Tiekink

**Affiliations:** 1 Medical Department Pharmachemie B. V. Haarlem NL-2003 The Netherlands; 2 Department of Chemistry National University of Singapore 117543 Singapore

## Abstract

A series of triorganophosphinegold(1) dithiocarbamate (R_3_PAuS_2_CNR'_2_) and xanthate (R_3_PAuS_2_COR') complexes have been prepared and characterised spectroscopically. Based on crystallographic evidence, the molecules feature linear gold(1) geometries defined by sulphur and phosphorus donors. The complexes, along with a series of known anti-cancer agents, have been screened against a panel of seven human cancer cell lines. Uniformly, the dithiocarbamate derivatives are more active than their xanthate counterparts, with the most active complex being Et_3_PAu(S_2_CNEt_2_), and are more active than *cisplatin* in all cell lines screened but, not as potent as *taxol*.


### Cytotoxicity Profiles for a Series of Triorganophosphinegold(I) Dithiocarbamates and Triorganophosphinegold(I) Xanthates

Dick de Vos[1], Soo Yei Ho[2] and Edward R.T. Tiekink[2,*]

[1] *Medical Department, Pharmachemie B.V., NL-2003, Haarlem, The Netherlands.*
[2] *Department of Chemistry, National University of Singapore, Singapore 117543; Tel.: 65 6874 2848; Fax.: 65 779 1691; e-mail: chmtert@nus.edu.sg*

### ABSTRACT

A series of triorganophosphinegold(I) dithiocarbamate ($R_3PAuS_2CNR'_2$) and xanthate ($R_3PAuS_2COR'$) complexes have been prepared and characterised spectroscopically. Based on crystallographic evidence, the molecules feature linear gold(I) geometries defined by sulphur and phosphorus donors. The complexes, along with a series of known anti-cancer agents, have been screened against a panel of seven human cancer cell lines. Uniformly, the dithiocarbamate derivatives are more active than their xanthate counterparts, with the most active complex being $Et_3PAu(S_2CNEt_2)$, and are more active than *cisplatin* in all cell lines screened but, not as potent as *taxol*.

**Keywords:** Gold, thiolate, phosphine, cytotoxicity, dithiocarbamate, xanthate

### INTRODUCTION

Amongst the 1,1-dithiolate ligands, dithiocarbamates, $^-S_2CNR_2$, comprise a group of ligands with great binding potential to metals and as such find wide use in coordination chemistry. Their synthesis is relatively simple with the most common method of preparation involving the reaction of carbon disulphide, in the presence of a base such as sodium or potassium hydroxide, with any one of a large range of primary and secondary amines /1, 2/. Dithiocarbamates and their metal complexes have a wide variety of applications. Their most common use is as pesticides, *e.g.* zineb, $[Zn(S_2CN(H)CH_2CH_2N(H)CS_2)]_n$, maneb, $[Mn(S_2CN(H)CH_2CH_2N(H)CS_2)]_n$, ziram, $Zn(S_2CNMe_2)_2$, and thiram, $Me_2NC(=S)SSC(=S)NMe_2$, and this application has led to the development of new analytical techniques that were designed to determine the concentrations of these pesticides as well as their degradation products /2 - 4/. In addition, these species have important applications in the production of petroleum derivatives, lubricants and polymers, where they are used as accelerators for vulcanization, anti-oxidants and anti-humidity agents /5 - 7/. Some dithiocarbamate ligands are excellent reagents for the analysis of trace metals by means of enhancement techniques /8/.

Dithiocarbamates are well known as heavy-metal chelating agents with a strong affinity for many divalent cations, as well as the heavier elements, and possess various biological activities. For example, their chelating abilities can cause the inhibition of numerous metal-containing enzymes, such as copper-containing dopamine-β-hydroxylase, superoxide dismutase (SOD), glutathione peroxidase, and cytochrome oxidase /9/.

An approved agent given to human patients with alcohol-abuse problems is disulfram (Antabuse®), *i.e.* $Et_2NC(=S)SSC(=S)NEt_2$ /10/. Diethyldithiocarbamate has had extensive clinical use in the treatment of Wilson's disease, *i.e.* copper poisoning, and a variety of other heavy-metal poisoning /11/. During the past decade, there has been considerable interest in the possible use of dithiocarbamate ligands in the treatment of cancer with ionizing radiation /12/. It is thought that diethyldithiocarbamate enhances radiation sensitivity owing to its inhibition of SOD, as mentioned above /12/. Over and above this, diethyldithiocarbamate has shown anti-cancer effects in its own right /9/. For example, diethyldithiocarbamate can reduce alkylation of DNA by nitrosamines /13/ and it can inhibit tumour induction by the cancer inducing agent benzo[α]pyrene /14/. Finally, dithiocarbamate has been shown to be effective in the reduction of several secondary effects associated with chemotherapeutic agents such as *cisplatin* /15/. Thus, nephrotoxicity may be reduced /16/ by its complexing with platinum-enzyme adducts formed in the kidney /17/ and myelosuppression may be moderated /18/. It is thought that it is the inhibition of diethyldithiocarbamate metabolism by the microsomal mixed oxygenase system that is responsible for its reduction of carcinogenic effects and toxic side-effects associated with chemotherapy /19/. Indeed, there is a suggestion that diethyldithiocarbamate is unique among potential *cisplatin* chemoprotectors in the selectivity of its reactions with *cisplatin* or more precisely, with its metabolites. Finally, diethyldithiocarbamate has been reported to inhibit progression of HIV implicated in AIDS /20, 21/. A related dithiocarbamate ligand, *i.e.* pyrrolidinedithiocarbamate, ($^{-}S_2CN(CH_2)_4$), is also of biological importance as it was found to inhibit NF-κB-related gene-expression /22/.

Among the various different classes of metal complexes currently investigated for their applications in medicine, dithiocarbamate complexes demonstrate outstanding potential /23/. The potential medical uses of dithiocarbamate complexes include: anti-viral agents, *e.g.* heterocyclic dithiocarbamates of ruthenium(III) /24/, antidotes for preventing the effects of phytotoxic agents, *e.g.* copper dithiocarbamates /25/, bactericides and anti-microbial agents, *e.g.* triorganotin dithiocarbamates /26/, anti-tumour agents, notably of palladium and platinum /27-31/ as well as tin dithiocarbamates /32, 33/, anti-parasitic agents, *e.g.* platinum, iridium and rhodium /34/, and prophylactic or therapeutic agents for metal toxicity, *e.g.* for cadmium /35/. Iron dithiocarbamates have been used for treating AIDS and neurodegenerative diseases /36/. As an extension of the aforementioned medical applications of dithiocarbamate ligands and their metal complexes, this contribution describes a study where dithiocarbamates have been combined with phosphinegold(I) entities with the view of exploring their anti-tumour potential.

Gold thiolates, including a phosphinegold(I) thiolate, auranofin, are used in the treatment of arthritis /37, 38/, usually after other therapies have been exhausted. The examination of the potential anti-tumour activity of gold complexes is a more recent phenomenon and has been demonstrated in a number of experimental models but, as yet, no gold compound has entered clinical trials. Auranofin and analogues were shown to be cytotoxic towards B16 melanoma and P388 leukaemia *in vivo*, early standards in anti-cancer screening /39/. The development of gold complexes as anti-tumour agents has been reviewed recently /40/.

The focus of our investigations in this field has been upon the potential anti-tumour activity of phosphinegold(I) thiolates, *i.e.* auranofin analogues /41/. A particular emphasis has been to couple biologically active thiols with phosphinegold(I) entities in the hope that upon administration of the 'pro-drug', both the phosphinegold(I) entity and thiol would provide therapeutic benefit /42, 43/. As a continuation upon this theme, we present here the results of *in vitro* cytotoxicity screening for a range of phosphinegold(I) dithiocarbamate complexes, a study motivated by the combination of biologically active dithiocarbamates with phosphinegold(I). In addition, a smaller number of phosphinegold(I) dithiocarbonate ($^{-}S_2COR$, xanthate) complexes are included in this study. Xanthates and their metal complexes have not been evaluated for biological activity to the same extent as dithiocarbamates. However, xanthate complexes of tin have demonstrated some potential as anti-tumour agents /44/ and certain phosphinegold(I) dithiocarbonate complexes have proved to possess some anti-arthritic activity /45/. The results of this study are reported herein.

### EXPERIMENTAL

### General

The $R_3PAuCl$ starting materials were prepared according to the literature method /46/. All solvents were of analytical grade (J. T. Baker) and used as supplied. $NaS_2CNEt_2.3H_2O$ (Tuka) and $NH_4S_2CNC_4H_8$ (Aldrich) were used as supplied. Potassium xanthates were prepared from the reaction of the alcohol (that also served as the solvent), $CS_2$ and KOH. $^1H$, and $^{13}C\{^1H\}$ NMR spectra were recorded on a Bruker ACF300 FT NMR spectrometer, with chemical shifts relative to tetramethylsilane. $^{31}P\{^1H\}$ NMR data were recorded on the same instrument but with chemical shifts recorded relative to 85% aqueous $H_3PO_4$. IR spectra were obtained as KBr pellets on a Bio-Rad FTS165 FTIR spectrophotometer. ESI mass spectra were measured on a Finnigan MAT95XL-T spectrometer. Elemental analyses were performed on a Perkin Elmer PE 2400 CHN Elemental Analyser.

#### *General Synthetic Procedure*

To a dichloromethane solution (4 ml) of $R_3PAuCl$ was added an equimolar amount (based on gold content) of dithiolate ligand. The colourless solution immediately turned yellow, indicating the formation of the product, and was stirred for 2 h. The yellow solution was filtered through Celite and concentrated to approximately 1 ml to yield the product.

#### *$Et_3PAuS_2CNEt_2$ (1)*

From $Et_3PAuCl$ (0.2 g, 0.57 mmol) and $NaS_2CNEt_2$ (98 mg, 0.57 mmol). The product was recrystallised by the layering of ethanol into a dichloromethane solution of the compound to yield yellow crystals. Yield: 200 mg (76 %). δ ($^{31}P\{^1H\}$, $CDCl_3$): 32.7 ppm; ESI-MS: m/z 778 ($2M^+$ - $S_2CNEt_2$).

***$Cy_3PAuS_2CNEt_2$ (2)***

From $Cy_3PAuCl$ (0.20 g, 0.39 mmol) and $NaS_2CNEt_2$ (67 mg, 0.39 mmol). The product was recrystallised by the vapour diffusion of hexane into a dichloromethane solution of the compound to yield yellow crystals /47/. Yield: 192 mg (79 %). δ ($^{31}P\{^1H\}$, $CDCl_3$): 55.3 ppm; ESI-MS: m/z 626 ($M^+$); 757 ($M^+$ + Au); 1103 ($M^+$ - $NEt_2)_2$.

***(p-MeOPh)$_3PAuS_2CNEt_2$ (3)***

From (p-MeOPh)$_3PAuCl$ (0.2 g, 0.34 mmol) and $NaS_2CNEt_2$ (67 mg, 0.39 mmol). The product was recrystallised by the vapour diffusion of methanol into a chloroform solution of the compound to yield yellow crystals /48/. Yield: 168 mg (71 %). δ ($^{31}P\{^1H\}$, $CDCl_3$): 32.5 ppm; ESI-MS: m/z 1247 ($M^+$ - $NEt_2)_2$.

***dppfAu$_2[S_2CNEt_2]_2$ (4)***

From dppfAu$_2Cl_2$ (0.1 g, 0.098 mmol) and $NaS_2CNEt_2$ (34 mg, 0.196 mmol). The product was recrystallised by the layering of ethanol into a dichloromethane solution to yield an orange solid. Yield: 75 mg (62 %). δ ($^{31}P\{^1H\}$): 30.5 ppm; ESI-MS: m/z 1096 ($M^+$ - $NEt_2)_2$)

***$Cy_3PAuS_2CNC_4H_8$ (5)***

From $Cy_3PAuCl$ (0.10 g, 0.20 mmol) and $NH_4S_2CNC_4H_8$ (32 mg, 0.20 mmol). The product was recrystallised by the vapour diffusion of hexane into a dichloromethane solution of the compound to yield yellow crystals /49/. Yield: 83 mg (67 %). δ ($^{31}P\{^1H\}$, $CDCl_3$): 55.4 ppm; ESI-MS: m/z 624 ($M^+$).

***$Ph_3PAuS_2CNC_4H_8$ (6)***

From $Ph_3PAuCl$ (0.10 g, 0.20 mmol) and $NH_4S_2CNC_4H_8$ (32 mg, 0.20 mmol) The product was recrystallised by the vapour diffusion of ethanol into a dichloromethane solution of the compound to yield yellow crystals. Yield: 86 mg (71 %). δ ($^{31}P\{^1H\}$): 36.2 ppm; ESI-MS: m/z 721 (($Ph_3P)_2Au$); 1064 ($2M^+$ - $S_2CNC_4H_8$).

***dppfAu$_2[S_2CNC_4H_8]_2$ (7)***

From dppfAu$_2Cl_2$ (0.10 g, 0.098 mmol) and $NaS_2CNC_4H_8$ (34 mg, 0.196 mmol) The product was recrystallized by the layering of ethanol into a dichloromethane solution to yield an orange solid. Yield: 82 mg (67 %). δ ($^{31}P\{^1H\}$): 27.8 ppm; ESI-MS: m/z 1095 ($M^+$ - $S_2CNC_4H_8$).

***$Ph_3PAuS_2COC_4H_9$ (8)***

From $Ph_3PAuCl$ (0.10 g, 0.20 mmol) and $KS_2COC_4H_9$ (33 mg, 0.220 mmol). The product was recrystallised by the vapour diffusion of ethanol into a dichloromethane solution of the compound to yield yellow crystals. Yield: 98 mg (81 %). δ ($^{31}P\{^1H\}$): 37.5 ppm; ESI-MS: m/z 721 (($Ph_3P)_2Au^+$); 1067 ($2M^+$ - $S_2COC_4H_9$).

***$Ph_3PAu$ $S_2COCH_2CH_2OMe$ (9)***

From $Ph_3PAuCl$ (0.10 g, 0.20 mmol) and $KS_2COCH_2CH_2OMe$ (33 mg, 0.20 mmol). The product was recrystallised by the vapour diffusion of ethanol into a dichloromethane solution of the compound to yield yellow crystals. Yield: 87 mg (71 %). δ ($^{31}P\{^1H\}$): 33.3 ppm; ESI-MS: m/z 721 ($(Ph_3P)_2Au^+$); 1069 ($2M^+$ - $S_2COCH_2CH_2OMe$).

***(p-MeOPh)$_3$PAuS$_2$CO$^i$Pr (10)***

From (p-MeOPh)$_3$PAuCl (0.10 g, 0.17 mmol) and $KS_2CO^iPr$ (26 mg, 0.17 mmol). The product was recrystallized by the vapour diffusion of methanol into a chloroform solution of the compound to yield yellow crystals. Yield: 86 mg (74 %). δ ($^{31}P\{^1H\}$): 33.7 ppm; ESI-MS: m/z 901 ($((p\text{-}CH_3OPh)_3P)_2Au^+$); 1233 ($2M^+$ - $S_2CO^iPr$).

### Crystallography

X-ray data for (p-MeOPh)$_3$PAuS$_2$CO$^i$Pr (**10**) were collected on a Bruker AXS SMART CCD diffractometer using Mo-Kα radiation at 183 K so that $\theta_{max}$ was 30.0°. Data were reduced (SMART & SAINT /50/) and corrected for absorption effects (SADABS /51/). The structure was solved by heavy-atom methods (PATTY in DIRDIF /52/) and refined (anisotropic displacement parameters, H atoms in calculated positions, and a weighting scheme of the form $w = 1/[\sigma^2(F_o^2) + 0.0051P^2 + 0.4062P]$ where $P = (F_o^2 + 2F_c^2)/3$) on $F^2$ (SHELXL-97 /53/). Crystallographic data: $C_{25}H_{28}AuO_4PS_2$, $M$ = 684.53, orthorhombic, space group $Pna2_1$, $a$ = 12.3140(6), $b$ = 12.2750(6), $c$ = 17.2707(8) Å, $V$ = 2610.5(2) Å$^3$, $Z$ = 4, $D_x$ = 1.742 g cm$^{-3}$, 20822 reflections measured, 7397 unique ($R_{int}$ = 0.041) and 6683 with $I \geq 2\sigma(I)$. $R$ (obs. data) = 0.033 and $wR$ = 0.080 (all data). $\rho_{max}$ = 1.90 e Å$^{-3}$ (near Au). Flack parameter /54/ = -0.010(6). The molecular structure showing the atomic numbering scheme is shown in Fig. 1 (50% displacement ellipsoids, ORTEP /55/). Data manipulation was conducted with teXsan /56/. The CCDC deposition number is 217204.

### Cytotoxicity Screening

The test and reference compounds were dissolved to a concentration of 250 000 ng/ml in full medium, by 20 fold dilution of a stock solution which contained 1 mg compound/200 µl. The trial complexes (**1**) – (**10**) were taken into DMSO. However, it was noted that (**2**), (**4**) and (**5**) did not dissolve completely, even when heated to 60 °C. Cytotoxicity was estimated by the microculture sulforhodamine B (SRB) test /57/. The human cancer cell lines examined in the present study were: A498, renal cancer; MCF-7, estrogen receptor (ER)+/progesterone receptor (PgR)+; EVSA-T, estrogen receptor (ER)-/progesterone receptor (PgR)-; H226, non-small cell lung cancer; IGROV, ovarian cancer; M19 MEL, melanoma; and WIDR, colon cancer.

The experiment was started on day 0. On day 0, 150 µl of trypsinized tumor cells (1500 – 2000 cells/well) were plated in 96-wells flatbottom microtiter plates (falcon 3072, DB). The plates were preincubated 48 hrs at 37 °C, 8.5 % $CO_2$ to allow the cells to adhere. On day 2, a three-fold dilution sequence of ten steps was made in full medium, starting with the 250 000 ng/ml stock solution. Every dilution was used in quadruplicate by adding 50 µl to a column of four wells. This results in a highest concentration of 62 5000 ng/ml present in column 12. Column 2 was used for the blank. To column 1, PBS was added to diminish interfering evaporation. On day 7, the incubation was terminated by washing the plate twice with PBS. Subsequently, the cells were fixed with 10 % trichloroacetic acid in PBS and placed at 4 °C for one hour.

After five washings with tap water, the cells were stained for at least 15 minutes with 0.4 % SRB dissolved in 1 % acetic acid. After staining, the cells were washed with 1 % acetic to remove the unbound stain. The plates were air-dried and the bound stain was dissolved in 150 μl 10 mM Tris-base. The absorbance was read at 540 nm using an automated microplate reader (Labsystems Multiskan MS). Data were used for construction of concentration-response curves and determination of the $ID_{50}$ value by use of Deltasoft 3 software.

### RESULTS AND DISCUSSION

A series of phosphinegold(I) 1,1-dithiolates have been prepared and characterised spectroscopically. Physical data are presented in Table 1 and the spectroscopic results, that confirm the formation of the complexes, are summarised in Tables 2 - 4. The crystal structure of a representative complex has been undertaken.

The molecular structure of ($p$-$MeOC_6H_4)_3PAu(S_2COiPr)$ (**10**) is shown in Fig. 1 and selected geometric parameters are collected in the caption to this figure. The gold atom exists in the expected linear geometry defined by S and P donor atoms with the Au-S distance being significantly longer than the Au-P distance. The small deviation from the ideal linear angle at gold (S-Au-P is 176.75(5)°) may be traced to the close approach of the non-coordinating S2 atom that is separated 3.2856(16) Å from the gold atom. Arguably the most significant intermolecular contacts are of the type C-H...O. Thus, C16-H...O2$^{i}$ is 2.47 Å, C16...O2$^{i}$ is 3.319(6) Å and the angle at H is 149°, and C17-H...O3$^{ii}$ is 2.37 Å, C17...O3$^{ii}$ is 3.292(5) Å and the angle at H is 163° for symmetry operations *i*: 1-*x*, -*y*, ½+*z* and *ii*: -½+*x*, -½-*y*, *z*. Similar coordination geometries have been reported for related phosphinegold(I) xanthates /59 - 65/ and there is no evidence to suggest that different structures are found for (**8**) and (**9**). The molecular geometry found for (**10**) is also as expected for their phosphinegold(I) dithiocarbamate analogues /58/ and indeed, the crystal structures of (**2**) /47/, (**3**) /48/ and (**5**) /49/ have been reported separately.

**Table 1**

Physical data for $R_3PAuSR$

| | | | Found (%) | | Requires (%) | |
|---|---|---|---|---|---|---|
| Complex | State | M.p (°C) | C | H | C | H |
| $Et_3PAuS_2CNEt_2$ (**1**) | Yellow | 91-92 | 28.7 | 5.4 | 28.5 | 5.4 |
| $Cy_3PAuS_2CNEt_2$ (**2**) | Yellow | 188-189 | 44.3 | 7.1 | 44.2 | 6.9 |
| ($p$-$MeOPh)_3PAuS_2CNEt_2$ (**3**) | Yellow | 131 | 44.0 | 4.4 | 43.9 | 4.1 |
| $dppfAu_2[S_2CNEt_2]_2$ (**4**) | Orange | 192 | 42.5 | 4.0 | 42.5 | 3.9 |
| $Cy_3PAuS_2CNC_4H_8$ (**5**) | Yellow | 222-224 | 43.8 | 6.1 | 44.7 | 6.6 |
| $Ph_3PAuS_2CNC_4H_8$ (**6**) | Yellow | 183-184 | 45.6 | 3.8 | 46.1 | 3.8 |
| $dppfAu_2[S_2CNC_4H_8]_2$ (**7**) | Orange | 212 | 41.0 | 3.5 | 42.6 | 3.6 |
| $Ph_3PAuS_2COC_4H_9$ (**8**) | Yellow | 118-119 | 45.3 | 3.7 | 45.4 | 3.6 |
| $Ph_3PAu\ S_2COCH_2CH_2OMe$ (**9**) | Yellow | 150-151 | 43.2 | 3.9 | 43.2 | 3.8 |
| ($p$-$MeOPh)_3PAuS_2CO^iPr$ (**10**) | Yellow | 110-111 | 44.0 | 4.4 | 44.0 | 4.4 |

The phosphinegold(I) dithiocarbamates, (**1**) – (**7**), and xanthates, (**8**) – (**10**), have been evaluated for their cytotoxicity against a panel of seven human cancer cell lines. The following cell lines were used: A498, renal cancer; MCF-7, estrogen receptor (ER)+/progesterone receptor (PgR)+; EVSA-T, estrogen receptor (ER)-/progesterone receptor (PgR)-; H226, non-small cell lung cancer; IGROV, ovarian cancer; M19 MEL, melanoma; and WIDR, colon cancer. The A498, H226, IGROV, M19 MEL, WIDR cell lines are included in the current anti-cancer screening panel of the National Cancer Institute, U.S.A. /66/. The cytotoxicity screening results for (**1**) – (**10**) are given in Table 5 as well as those for a series of standard anti-cancer agents. From the data presented in Table 5, several trends may be discerned.

The two dithiocarbamate ligands chosen for evaluation were featured in the *Introduction* owing to their known biological relevance. Amongst the diethyldithiocarbamates, the $Et_3P$ derivative (**1**) was the most active. The $Cy_3P$ species (**2**) has comparable cytotoxicity to (**1**) and both are more potent than the (*p*-$MeOC_6H_4)_3P$ derivative (**3**). The complex containing the bidentate phosphine ligand dppf, where dppf is 1,1'-bis(diphenylphosphine)ferrocene, that gives rise to a dinuclear gold species (**4**), has the poorest cytotoxicity, in particular considering it contains approximately twice the amount of gold as do the other species. The second dithiocarbamate series contains the pyrrolinedithiocarbamate ligand. Of the three complexes, (**5**) – (**7**), the $Ph_3P$ species (**6**) is the most potent. The $Cy_3P$ complex (**5**) is less cytotoxic against all cell lines compared with the diethyldithiocarbamate analogue (**2**) but the reverse is true for the dppf derivatives in five cell lines, *i.e.* A498, MCF-7, EVSA-T, M19 and WIDR. Such a non-systematic variation underscores the difficulty in generating a structure/activity relationship in these compounds. Amongst the xanthate, $Ph_3PAu(S_2COR)$, complexes, R = $(CH_2)_3CH_3$ (**8**) and $CH_2CH_2OCH_3$ (**9**) had comparable potency to each other and both were more cytotoxic than (*p*-$MeOC_6H_4)_3PAu(S_2COiPr)$ (**10**). As a class of complex, the xanthates are generally less cytotoxic than their dithiocarbamate analogues. The greatest potency exhibited by the xanthate complexes was against the ovarian cancer cell line IGROV but, it is noted that the range of $ID_{50}$ values against all cell lines is not great suggesting little, if any, specificity in their cytotoxicity profile. The greatest potency exhibited by the dithiocarbamate complexes was also evident against the IGROV cell line and comparable activities were also found against the breast cancer cell lines MCF-7 and EVSA-T. The cytotoxicity results for the phosphinegold(I) 1,1-dithiolates can be compared with those obtained for a selection of known anti-cancer agents.

**Table 2**

$^{1}$H NMR data (ppm, Hz) for phosphinegold(I) 1,1-dithiolates

| Complex | Thiolate ligand | | | | Phosphine ligand | | |
|---|---|---|---|---|---|---|---|
| (1) | 3.91q<br>$CH_2$<br>(7.2)$^{B}$ | 1.34t<br>$CH_3$<br>(7.2)$^{B}$ | | | 1.83dq<br>$CH_2$<br>(19.5)$^{A}$ | 1.22dt<br>$CH_3$<br>(18)$^{A}$ | |
| (2) | 3.91q<br>$CH_2$<br>(7.2)$^{B}$ | 1.30t<br>$CH_3$<br>(7.2)$^{B}$ | | | 2.02-1.54<br>Cy | | |
| (3) | 3.93q<br>$CH_2$<br>(7.2)$^{B}$ | 1.33t<br>$CH_3$<br>(7.2)$^{B}$ | | | 7.57-7.49<br>Ph | 6.95-6.91<br>Ph | 3.82s<br>$CH_3$ |
| (4) | 3.97q<br>$CH_2$<br>(7.2)$^{B}$ | 1.36t<br>$CH_3$<br>(7.2)$^{B}$ | | | 7.60-7.34<br>Ph | 4.9s<br>Fc | 4.3s<br>Fc |
| (5) | 3.83t<br>$CH_2$<br>(3.2)$^{B}$ | 1.99t<br>$CH_2$<br>(3.2)$^{B}$ | | | 2.18-1.72<br>Cy | | |
| (6) | 3.87t<br>$CH_2$<br>(6.8)$^{B}$ | 2.02tt<br><br>(3)$^{B}$ | | | 7.64-7.42<br>Ph | | |
| (7) | 3.84t<br>$CH_2$<br>(6.8)$^{B}$ | 2.08tt<br><br>(3)$^{B}$ | | | 7.61-7.45<br>Ph | 4.74s<br>Fc | 4.30s<br>Fc |
| (8) | 4.49t<br><br>$CH_2$<br>(6.8)$^{B}$ | 1.74<br>quintet<br>$CH_2$<br>(7.2)$^{B}$ | 1.41<br>sextet<br>$CH_2$<br>(7.2)$^{B}$ | 0.87t<br><br>$CH_3$<br>(7.6)$^{B}$ | 7.51-7.45<br><br>Ph | | |
| (9) | 4.65t<br>$CH_2$<br>(4.8)$^{B}$ | 3.72t<br>$CH_2$<br>(4.8)$^{B}$ | 3.33s<br>$CH_3$ | | 7.55-7.46<br>Ph | | |
| (10) | 1.49-1.41<br>CH | 1.39d<br><br>$CH_3$<br>(6.4) | | | 7.51-7.28<br><br>Ph | 6.99-6.95<br><br>Ph | 3.85s<br><br>$CH_3$ |

$^{A}$ *J*(P-H), $^{B}$ *J*(H-H)

**Table 3**

$^{13}$C NMR data (ppm, Hz) for phosphinegold(I) 1,1-dithiolates [A]

| Complex | Thiolate | | | | Phosphine | | | | |
|---|---|---|---|---|---|---|---|---|---|
| (1) | 49.1<br>$CH_2$ | 12.2<br>$CH_3$ | | | 18.5<br>$CH_2$<br>(33.8) | 8.9<br>$CH_3$ | | | |
| (2) | 48.9<br>$CH_2$ | 12.1<br>$CH_3$ | | | 33.5-<br>25.9<br>Cy | | | | |
| (3) | 63.0<br>$OCH_3$ | 55.3<br>$CH_2$ | 12.1<br>$CH_3$ | | 175.4<br>Cδ | 135.5<br>Cα<br>(15.1) | 114.6<br>Cβ<br>(7.5) | 104.8<br>Cγ | |
| (4) | 49.3<br>$CH_2$ | 12.3<br>$CH_3$ | | | 133.5<br>Cα<br>(14.2) | 131.1<br>Cγ | 128.7<br>Cβ<br>(12.0) | 75.6<br>Cβ on<br>Fc | 74.9<br>Cα on<br>Fc<br>(14.2) |
| (5) | 63.5<br>$CH_2$ | 54.1<br>$CH_2$ | | | 33.4<br>Cα<br>(27.3) | 30.5<br>Cδ | 27.2<br>Cγ<br>(11.2) | 26.1<br>Cβ<br>(14.4) | |
| (6) | 63.5<br>$CH_2$ | 54.3<br>$CH_2$ | | | 134.3<br>Cα<br>(13) | 131.3<br>Cγ | 129.0<br>Cβ<br>(11) | | |
| (7) | 54.3<br>$CH_2$ | 52.0<br>$CH_2$ | | | 133.5<br>Cα<br>(14.2) | 131.1<br>Cγ | 128.8<br>Cβ<br>(10.9) | 75.7<br>Cβ on<br>Fc<br>(7.6) | 74.9<br>Cα on<br>Fc<br>(10.9) |
| (8) | 74.2<br>$CH_2$ | 30.6<br>$CH_2$ | 19.3<br>$CH_2$ | 13.7<br>$CH_3$ | 134.2<br>Cα<br>(14) | 131.7<br>Cγ | 129.2<br>Cβ<br>(12) | | |
| (9) | 72.8<br>$CH_2$ | 70.1<br>$CH_2$ | 58.9<br>$CH_3$ | | 134.3<br>Cα<br>(14) | 131.3<br>Cγ | 129.2<br>Cβ<br>(12) | | |
| (10) | 55.4<br>CH | 30.9<br>$CH_3$ | | | 162.3<br>Cδ | 135.6<br>Cα<br>(16) | 114.7<br>Cβ<br>(13) | | |

[A] Numbering scheme: Cα- Cδ, atoms of P-bound substituents, with Cα being adjacent to the phosphorus. *J*(P-C) values in parentheses.

**Table 4**

Infrared data ($cm^{-1}$) for phosphinegold(I) 1,1-dithiolates

| Complex | ν(C-N)/ν(C-O)[A] | | ν(C-S) | | |
|---|---|---|---|---|---|
| (1) | 1495s | 1455s | 1074m | 1047m | 985m |
| (2) | 1476s | 1455s | 1083s | 991s | |
| (3) | 1499s | 1458s | 1105s | 1026s | 995s |
| (4) | 1495s | 1456s | 1099s | 1075s | 981s |
| (5) | 1445s | 1427s | 1002s | 953s | |
| (6) | 1480s | 1461s | 1100s | 1027m | 999s |
| (7) | 1461m | 1443s | 1099s | 1027s | 998s |
| (8) | 1193s[A] | 1157s[A] | 1100s | 1050s | |
| (9) | 1248m[A] | 1206m[A] | 1080s | 1000m | |
| (10) | 1260s[A] | 1200s[A] | 1087s | 1028s | |

[A] ν(C-O) for **(8)** - **(10)**

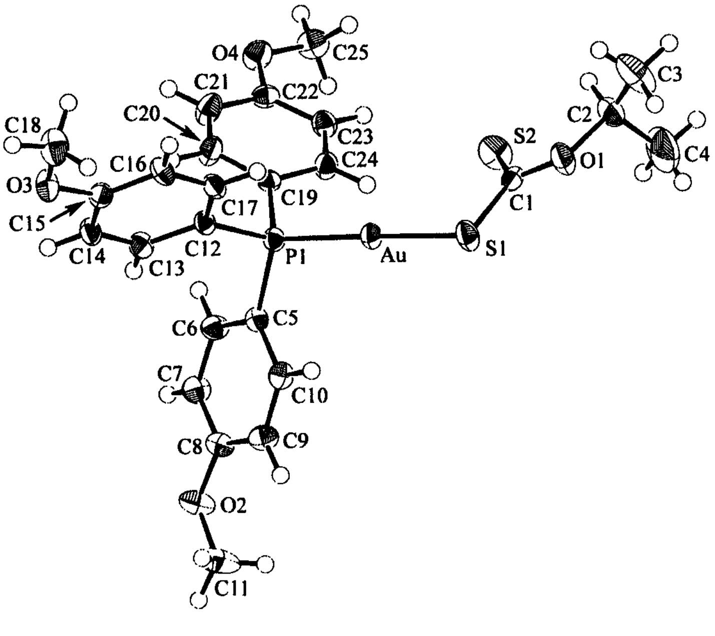

**Fig. 1:** Molecular structure and crystallographic numbering scheme for $(p\text{-MeOC}_6H_4)_3PAu(S_2COiPr)$ **(10)**. Selected geometric parameters: Au-S1 2.3159(11), Au-P1 2.2560(11), S1-C1 1.741(5), S2-C1 1.642(5), C1-O1 1.332(5), O1-C2 1.477(6) Å; S1-Au-P1 176.75(5), Au-S1-C1 100.17(16), S1-C1-S2, 125.9(3), S1-C1-O1 108.7(3), S2-C1-O2 125.4(3), C1-O1-C2 120.7(4)°.

It is clear from the data presented in Table 5 that several of the phosphinegold(I) dithiocarbamates and even xanthates had greater cytotoxicity than *cisplatin* in the cell lines evaluated. For the cell lines in which the dithiocarbamate complexes were particularly cytotoxic, *i.e.* IGROV, MCF-7 and EVSA-T, the $ID_{50}$ values were lower than those obtained for both 5-flurouracil and etoposide. Clearly, anti-cancer agents such as doxorubicin, methotrexate and taxol demonstrate greater cytotoxicity than the phosphinegold(I) 1,1-dithiolates.

**Table 5**

*In vitro* $ID_{50}$ values (ng/ml) for phosphinegold(I) 1,1-dithiolates and standard anti-cancer agents.[A]

| | Cell line | | | | | | |
|---|---|---|---|---|---|---|---|
| Complex | A498 | MCF-7 | EVSA-T | H226 | IGROV | M19 | WIDR |
| **(1)** | 196 | 21 | 16 | 45 | 12 | 83 | 141 |
| **(2)** | 213 | 39 | 42 | 69 | 26 | 197 | 339 |
| **(3)** | 529 | 84 | 98 | 134 | 58 | 303 | 288 |
| **(4)** | 2143 | 178 | 215 | 215 | 111 | 249 | 251 |
| **(5)** | 836 | 109 | 224 | 219 | 95 | 388 | 862 |
| **(6)** | 155 | 18 | 46 | 59 | 21 | 126 | 225 |
| **(7)** | 691 | 67 | 127 | 473 | 2196 | 201 | 114 |
| **(8)** | 934 | 972 | 435 | 1835 | 144 | 804 | 314 |
| **(9)** | 990 | 913 | 402 | 2365 | 208 | 825 | 355 |
| **(10)** | 2022 | 1257 | 854 | 2624 | 370 | 1394 | 522 |
| | | | | | | | |
| DOX | 90 | 10 | 8 | 199 | 60 | 16 | 11 |
| CPT | 2253 | 699 | 422 | 3269 | 169 | 558 | 967 |
| 5-FU | 143 | 750 | 475 | 340 | 297 | 442 | 225 |
| MTX | 37 | 18 | 5 | 2287 | 7 | 23 | < 3.2 |
| ETO | 1314 | 2594 | 317 | 3934 | 580 | 505 | 150 |
| TAX | < 3.2 | < 3.2 | < 3.2 | < 3.2 | < 3.2 | < 3.2 | < 3.2 |

[A] Abbreviations: Human cancer cell lines: A498, renal cancer; MCF-7, estrogen receptor (ER)+/progesterone receptor (PgR)+; EVSA-T, estrogen receptor (ER)-/progesterone receptor (PgR)-; H226, non-small cell lung cancer; IGROV, ovarian cancer; M19, melanoma; and WIDR, colon cancer. Standard anti-cancer agents: DOX, doxorubicin; CPT, cisplatin, 5-FU, fluorouracil; MTX, methotrexate; ETO, etoposide; and TAX, taxol.

### CONCLUSIONS

Phosphinegold(I) dithiocarbamates display cytotoxicity profiles greater than that exhibited by *cisplatin* against a range of human cancer cell lines. The most potent complex overall was $Et_3PAu(S_2CNEt_2)$ which was most active against the IGROV (ovarian cancer) cell line. The dithiocarbamate complexes had greater potency than the corresponding xanthate complexes.

### ACKNOWLEDGEMENTS

This research was supported by the National University of Singapore (R-143-00-139-112).

### REFERENCES

1 R. Soliman and H. M. Fiad-Ailah, *Egypt. J. Chem.*, **31**, 175 (1990)
2 C. C. Lo, M. H. Ho and M. D. Hung, *J. Agric. Food. Chem.*, **44**, 2227 (1996)
3 S. Castro, M. Vinocur, M. Permigiani, C. Halle, T. Taurian and A. Fabra, *Biol. Fert. Soils*, **25**, 147 (1997)
4 D. J. Johnson, D. G. Graham, V. Amarnath, K. Amarnath and W. M. Valentine, *Chem. Res. Toxicol.*, **9**, 910 (1996)
5 N. Segovia, G. Crovetto, P. Lardelli and M. Espigares, *J. Appl. Toxicol.*, **22** , 353 (2002)
6 S. Jiang, S. Dasgupta, M. Blanco, R. Frazier, E. S. Yamaguchi, Y. Tang and W. A. Gottard III, *J. Phys. Chem.*, **100**, 15760 (1996)
7 Y. Zhou, S. Jiang, T. Cagun, E. S. Yamaguchi, R. Frazier, A. Ho, Y. Tang and W. A. Goddard III, *J. Phys. Chem. A*, **104**, 2508 (2000)
8 D. Okatavec, J. Lehotay, V. Vrabel and E. Korgova, *Collect. Czech. Chem. Commun.*, **61**, 5 (1996)
9 A. Spath and K. Tempel, *Chem.-Biol. Interactions*, **64**, 151 (1987)
10 S. K. Delap, J. K. Skeeles, D. L. Kreider, C. E. Whitfill, J. N. Beasley, G. E. Houghten, E. M. Walker and D. J. Cannon, *Avian Disease*, **33**, 8 (1989)
11 F. W. Sunderman, *Ann. Clin. Lab. Sci.*, **9**, 1 (1979).
12 H. Wagner Jr, D. R. Parkinson, H. Madoc-Jones, E. S. Sternick, K. Vrusho and F. Krasin, *Radiation Oncol.*, **10**, 1575 (1984)
13 T. S. Ying, D. S. R. Sarma and E. Farber, *Am. J. Pathol.*, **99**, 159 (1980)
14 R. Grafstroem and F. E. Greene, *Biochem. Pharmacol.* **29**, 1517 (1980)
15 J. D. Khandekar, *Res. Commun. Chem. Path. Pharmacol.*, **40**, 55 (1983)
16 D. L. Bodenner, P. C. Dedon, P. C. Keng, J. C. Katz and R. F. Borch, *Cancer Res.*, **46**, 2745 (1986)
17 T. K. Schmalbach and R. F. Borch, *Cancer. Res.*, **49**, 6629 (1989)
18 D. L. Bodenner, P. C. Dedon, P. C. Keng, J. C. Katz and R. F. Borch, *Cancer Res.*, **46**, 2751 (1986)
19 Y. Masuda and N. Nakayama, *Toxicol. Appl. Pharmacol.*, **71**, 42 (1983)

20 R. Schreck, R. Grassmann, B. Fleckenstein and P. A. Baeuerle, *J. Virol.*, **66**, 6288 (1992)

21 J. L. Shenep, W. T. Hughes, P. M. Flynn, P. K. Roberson, F. G. Behm, G. H. Fullen, S. G. Kovnar, K. P. Guito and T. O. Brodkey, *Antimicrobial Agents Chemoth.*, **38**, 1644 (1994)

22 E. C. Reisinger, P. Kern, M. Ernst, P. Bock, H. D. Flad and M. Dietrich, *Lancet*, **335**, 679 (1990)

23 J. J. Criado, J. A. Lopez-Arias and B. Macias, *Inorg. Chim. Acta*, **193**, 229 (1992)

24 R. Ramesh and K. Natarajan, *Synth. React. Inorg. Met. Org. Chem.*, **26**, 1677 (1996)

25 I. Rogachev, V. Kampel, V. Gusis, N. Cohen, J. Gressel and A. Warshawasky, *Pest. Biochem. Physiol.* **60**, 133 (1998)

26 T. N. Srivastava and R. B. Rastogi, *Ind. J. Exp. Biol.*, **17**, 710 (1979)

27 R. Mital, N. Jain and T. S. Srivastava, *Inorg. Chim Acta*, **166**, 135 (1989)

28 L. Marcheselli, C. Preti, M. Tagliazucchi, V. Cherchi, L. Sindellari, A. Furlani, A. Papaioannou and V. Scarcia, *Eur. J. Med. Chem.*, **28**, 347 (1993)

29 G. Faraglia, D. Fregona, S. Sitran, L. Giovagnini, C. Marzano, F. Baccichetti, U. Casellato and R. Graziani, *J. Inorg. Biochem.*, **83**, 31 (2001)

30 C. Marzano, A. Trevisan, L. Giovagnini and D. Fregona, *Toxicol. Vitro*, **16**, 413 (2002)

31 D. Fregona, L. Giovagnini, L. Ronconi, C. Marzano, A. Trevisan, S. Sitran, B. Biondi and F. Bordin, *J. Inorg. Biochem.*, **93**, 181 (2003)

32 M.Carrara, L. Cima, S. Zampiron, C. Preti, V. Cherchi and L. Sindellari, *Anticancer Res.*, **9**, 775 (1989)

33 J. M. Hook, B. M. Linahan, R. L. Taylor, E. R. T. Tiekink, L. van Gorkom and L. K. Webster, *Main Group Metal Chem.*, **17**, 293 (1994)

34 S. L. Croft, R. A. Neal, D. G. Craciunescu and G. Certad Fombard, *Trop. Med. Parasitol.*, **43**, 24 (1992)

35 M. M. Jones and M. G. Chevian, *Toxicol.*, **62**, 1 (1990)

36 R. Schreck, B. Meiser, D. N. Mannel, W. Droege and P. A. Baeuerle, *J. Exp. Med.*, **175**, 1181 (1992)

37 A. J. Lewis and D. T. Walz, *Prog. Med. Chem.*, **19**, 1 (1982)

38 D. R. Haynes and M. W. Whitehouse, in K. D. Rainsford and G. P. Velo, Editors: *New Developments in Antirheumatic Therapy. Inflammation and Drug Therapy Series.* Volume III. Kluwer Academic Publishers, Dordrecht, 207 (1989)

39 C. K. Mirabelli, R. K. Johnson, D. T. Hill, L.F. Faucette, G. R. Girard, G. Y. Kuo, C. M. Sung and S. T. Crooke, *J. Med. Chem.* **29**, 218 (1986)

40 E. R. T. Tiekink, *Crit. Rev. Oncol./Hematology*, **42**, 225 (2002)

41 E. R. T. Tiekink, *Bioinorg. Chem. Applns*, **1**, 53 (2003)

42 E. R. T. Tiekink, P. D. Cookson, B. M. Linahan and L. K. Webster, *Metal-Based Drugs*, **1**, 299 (1994)

43 L. K. Webster, S. Rainone, E. Horn and E. R. T. Tiekink, *Metal-Based Drugs*, **3**, 63 (1996)

44 N. Donoghue, E. R. T. Tiekink and L. Webster, *Appl. Organomet. Chem.* 7, 109, (1993)

45 M. W. Whitehouse, P. D. Cookson, G. Siasios and E. R. T. Tiekink, *Metal-Based Drugs*, **4**, 245 (1998)

46 A. K. Al-Saády, C. A. McAuliffe, R. V. Parish and J. A. Sandbank, *Inorg. Synth.*, **23**, 191 (1985)

47 S. Y. Ho and E. R. T. Tiekink, *Acta Crystallogr.*, E57, m603 (2001)

48 S. Y. Ho and E. R. T. Tiekink, *Acta Crystallogr.*, **E58**, m86 (2002)

49 S. Y. Ho and E. R. T. Tiekink, *Z. Kristallogr. NCS*, **217**, 359 (2002)

50 SMART & SAINT Software Reference manuals, Version 5.0, Bruker AXS Inc., Madison, WI, 1998

51 G. M. Sheldrick, SADABS software for empirical absorption correction, University of Göttingen, Germany (2000)

52 P. T. Beurskens, G. Admiraal, G. Beurskens, W. P. Bosman, S. García-Granda, J. M. M. Smits, C. Smykalla, The DIRDIF program system, Technical Report of the Crystallography Laboratory, University of Nijmegen, The Netherlands (1994)

53 G. M. Sheldrick, SHELXL-97. Program for the refinement of crystal structures. University of Göttingen, Germany (1997)

54 H. D. Flack, *Acta Crystallogr.* **A39**, 876 (1983)

55 C. K. Johnson, ORTEP-II, Report ORNL-5138, Oak Ridge National Laboratory, Oak Ridge, Tennessee (1976)

56 teXsan, Structure Analysis Package, Molecular Structure Corporation, Woodlands, Texas (1992)

57 Y. P. Keepers, P. E. Pizao, G. J. Peters, J. van Ark-Otte, B. Winograd and H. M. Pinedo, *Eur. J. Cancer*, **27**, 897 (1991)

58 J. G. Wijnhoven, W. P. Bosman and P. T. Beurskens, *J. Cryst. Mol. Struct.*, **2**, 7 (1972)

59 E. R. T. Tiekink, *Z. Kristallogr.*, **173**, 243 (1985)

60 G. Siasios and E. R. T. Tiekink, *Z. Kristallogr.*, **198**, 139 (1993)

61 G. Siasios and E. R. T. Tiekink, *Z. Kristallogr.*, **203**, 117 (1993)

62 G. Siasios and E. R. T. Tiekink, *Z. Kristallogr.*, **204**, 95 (1993)

63 G. Siasios and E. R. T. Tiekink, *Z. Kristallogr.*, **205**, 261 (1993)

64 A. J. Collins and E. R. T. Tiekink, *Z. Kristallogr.*, **210**, 51 (1995)

65 S. Y. Ho and E. R. Tiekink, *Acta Crystallogr.*, **E59**, m53 (2003)

66 M. R. Boyd, *Prin. Pract. Oncol.*, **3**, 1, (1989)

